# Systematically Exploring Molecular Aggregation and Its Impact on Surface Tension and Viscosity in High Concentration Solutions

**DOI:** 10.3390/molecules25071588

**Published:** 2020-03-30

**Authors:** Huan Wang, Han Kong, Jie Zheng, Hui Peng, Chuangui Cao, Yong Qi, Kuanjun Fang, Weichao Chen

**Affiliations:** College of Textiles & Clothing, State Key Laboratory for Biofibers and Eco-textiles, Collaborative Innovation Center for Eco-textiles of Shandong Province, Qingdao University, Qingdao 266071, China; wh17861439837@163.com (H.W.); konghan19990316@163.com (H.K.); zhengjie2009123@126.com (J.Z.); Penghui7426@163.com (H.P.); c17669490708@163.com (C.C.); qy18865731238@163.com (Y.Q.)

**Keywords:** aggregation, viscosity, surface tension, high concentration, dyes

## Abstract

The aggregation structure of dye molecules has a great influence on the properties of dye solutions, especially in high concentration. Here, the dye molecular aggregation structures were investigated systemically in aqueous solutions with high concentration using three reactive dyes (O-13, R-24:1 and R-218). O-13 showed stronger aggregation than R-24:1 and R-218. This is because of the small non-conjugate side chain and its β-linked position on the naphthalene of O-13. Compared with R-218, R-24:1 showed relatively weaker aggregation due to the good solution of R-24:1. The change of different aggregate distributions in the solutions were also investigated by splitting the absorption curves. Moreover, it is found that the surface tension of solutions can be modified by the combined effect of both aggregation and the position of the hydrophilic group, which, however, also have an effect on viscosity. This exploration will provide guidance for the study of high concentration solutions.

## 1. Introduction

The aggregation behavior of dye molecules is common in aqueous solutions because of molecular interaction [1,2,3,4]. The aggregation has crucial influences on properties of solutions, especially highly concentrated solutions, which are usually used as raw materials for the industrial production processes such as fiber dyeing and printing and photoelectric thin film preparation [5,6,7,8,9,10,11,12,13,14,15]. Meanwhile, the surface tension and viscosity of the solutions are also important factors determining the properties of dye solutions [16], which would be affected by the dye molecular aggregation structure [17]. Therefore, it is necessary to investigate the aggregation structure and explore its relationship with surface tension and viscosity in high concentration dye solutions systematically using a simple model.

Reactive dyes, one kind of important organic dyes, are mainly used for dyeing and printing natural fabrics [18]. These dye molecules contain water-soluble groups (usually sodium sulfonate), and have good water solubility [19], but the dye molecules still tend to aggregate by self-association of the hydrophobic π-π conjugated parts of the molecular structure [20,21,22,23,24], which will lead to the formation of dimers, trimers [25], etc. The aggregation of dyes dilute solution has been investigated by many methods, such as, polarography [26,27], conductometry [28,29], UV-Vis [30,31,32,33], NMR [34], light scattering [35,36,37] measurements. The UV-Vis absorption spectroscopy is one of the most used methods for studies of the aggregation process [38,39,40]. Traditionally, dye aggregates are classified as H and J-type (blue-shifted and red-shifted) by observing the change of the aggregate peak relative to the position of its monomer absorption peak [41,42,43]. Therefore, aggregates in solution usually have significant changes in their absorption spectrum in comparison to the monomers.

However, the aggregation in concentrated dye solutions is difficult to investigate by the UV-Vis method since the absorbance of concentrated solutions often exceeds the measured limits of the instrument. Recently, the appearance of cuvette with short optical path of 0.01 mm instead of the regular 10 mm makes it possible to study the aggregation of highly concentrated dye solutions with UV-Vis method.

In this work, three kinds of the most commonly used triazine reactive dyes, C.I. Reactive Orange 13 (O-13), C.I. Reactive R218 (R-218), and C.I. Reactive Red 24:1 (R-24:1), were chosen to investigate their aggregation behavior in the highly concentrated solutions. The change of aggregation structures with the different concentrations were explored by simply splitting the UV-Vis absorption curves. In order to make sure the absorbance was not beyond the detection limit, cuvettes with different short path lengths were used. It was found that the dyes’ aggregation was affected by the non-conjugate part of the dyes and their linked position on the naphthalene (α or β). Meanwhile, solubility was also important for dyes aggregation. The distribution of different aggregates in the solutions could be obtained. Mover, it is found that the surface tension can be modified by both aggregation and the position of hydrophilic group.

## 2. Results and Discussion

### 2.1. Molecular Aggregation of High Concentration Solutions

#### 2.1.1. Absorption Spectra of the Three Dyes Solutions

**Figure 1:**
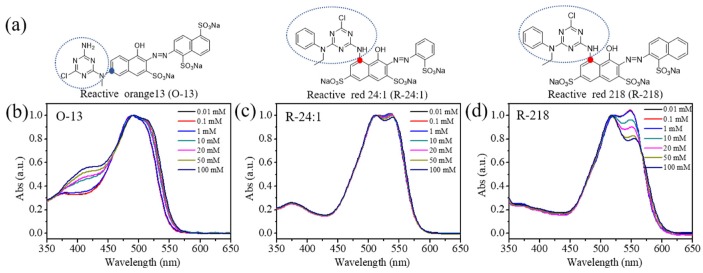
(**a**) The molecular structures of the three dyes (O-13, R-24:1 and R-218), (**b**–**d**) the normalized absorption spectra of the three dyes under different concentrations, respectively.

The UV-Vis absorption spectra of reactive dye solutions of different concentrations of 10^−3^ mM to 10^2^ mM was measured using cuvettes with different optical paths ranging from 0.01 mm to 10 mm and results showed that the spectra of highly concentrated solutions could only be obtained with short optical paths’ cuvettes, which was in accordance to the Langer-beer’s law. The normalized absorption spectra of solutions with concentrations of 0.01, 0.1, 1, 10, 20, 50, and 100 mM were shown in Figure 1b–d for three dyes. Three kinds of dye solutions showed different trends with the increase of dye concentrations. The two main peaks in the long wavelength come from the monomer and dimer absorption, respectively. As we know, the dye molecules exist in aqueous solution in the form of H aggregation [44,45,46,47]. Therefore, the longer wavelength peak belongs to monomer absorption, and the slightly shorter peak comes from the dimer absorption. For O-13, with the increase of concentration, the two main peaks, one at 488 nm and one shoulder peak at 510 nm, had a slight redshift without obvious intensity changes. However, the intensity of peaks near 410 nm enhanced gradually with the increase of concentration. R-24:1 showed two main peaks at 512 and 536 nm without any appearance of new peaks, and the longer peak decreased slightly with the increase of concentration. Two main peaks were also found for R-218, but the longer peak decreased much more obviously with the increase of concentration (especially over 1 mM). The different trends of changes for three dyes might be caused by the difference of their molecular structures.

#### 2.1.2. Molecular Structure of the Dye Molecules

**Figure 2:**
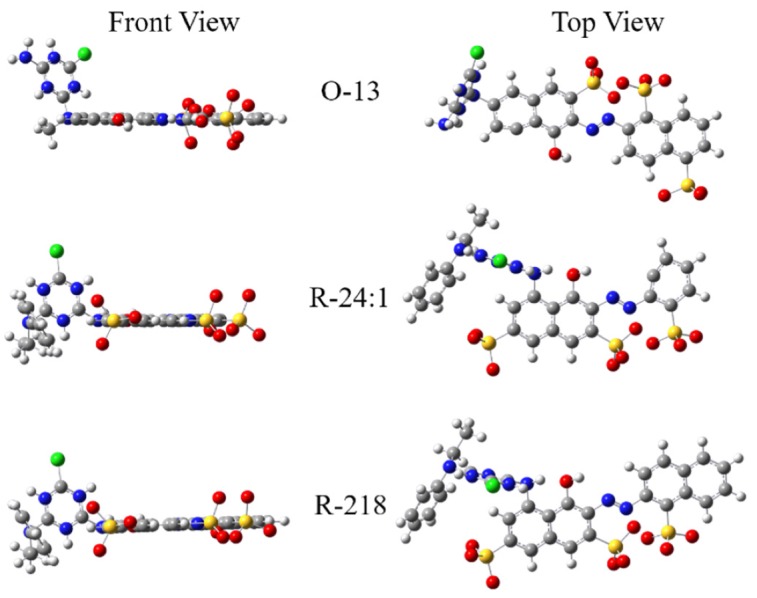
The stable molecular structures of the three dyes.

The stable molecular structural formula of the three dyes were simulated by theoretical simulation, which were performed with the density functional theory (DFT) method using the GAUSSIAN 09 package [48,49,50]. The ground-state geometries of all species were optimized using the B3LYP functional 33–35 with the 6–31 + G* basis set. As shown in Figure 2, all three dyes have very flat conjugate parts, while O-13 has a relatively smaller non-conjugated part than R-24:1 and R-218, which have the same non-conjugated part. Meanwhile, the non-conjugated parts of R-24:1 and R-218 are both linked to the α-position of naphthalene ring while that of O-13 is linked to the β-position of the naphthalene ring. Since the β-position was relatively further away from the conjugated part than the α-position, the non-conjugated part of O-13 has less effect on the molecular stacking. This may be an important cause for the enhanced aggregation for O-13. Compared with R-24:1, R-218 has the same number of water-soluble groups (three sodium sulfonate) but one more benzene ring in the conjugated part, indicating its worse solubility than R-24:1, which resulted in relatively larger changes than R-24:1 as the concentration increased, as shown in Figure 1.

#### 2.1.3. The Aggregation Distributions of the Three Dye Solutions

**Figure 3:**
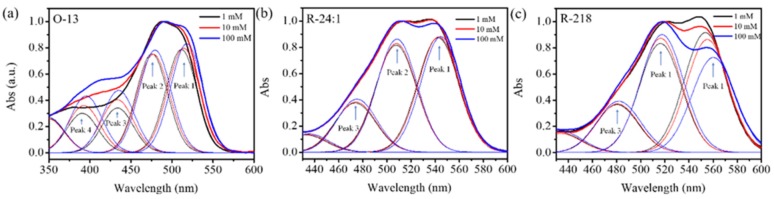
Deconvoluted absorption spectra of (**a**) O-13, (**b**) R-24:1 and (**c**) R-218 dyes absorption spectra under different concentrations.

The change of aggregation states for the three dyes as the increased concentration were also investigated by splitting the absorption spectra with a Gaussian curve fitting program, as shown in Figure 3. The sum of the fitting Gaussian curves should be consistent with the UV-Vis absorption curves [51,52]. It is found that 4 peaks and 3 peaks are suitable for the O-13 and R-24:1 and R-218 by multiple attempts to fit the data using Gaussian function, respectively. The peak1 and peak 2 of the Gaussian curves was consisted with the monomer and dimer absorption peaks of the absorption spectrum by comparing the Gaussian curves with the absorption spectrum, respectively. Therefore, the Gaussian peak 1 and peak 2 derive from monomer and dimer absorption respectively. The peak 3 and peak 4 may come from the absorption of higher aggregates. The ration of different aggregation in the solution was calculated by comparing the areas under each component curve with the total curve; the corresponding parameters were given in the Table 1.

It can be found that three dyes showed different variation trends as the concentration increased. For O-13, the rations of monomer and dimer absorption both decreased (from 33.2% to 30.6% for monomer absorption and from 31.8% to 28.8% for dimer absorption), and the multimolecular aggregates (corresponding to peaks 3 and 4) increased (from 14.4% to 17.5% for monomer and from 12.6% to 16.0% for dimer) with the enhanced concentration from 1 mM to 100 mM. This indicated that the intermolecular aggregation interaction may be occurred primarily in between monomer and dimer, as well as dimer aggregation interaction. The R-24:1 and R-218 showed different variation tendency with O-13. With the concentration increasing from 1 mM to 100 mM, the rations of monomer absorption for R-24:1 and R-218 both decreased, while the rations of dimer and higher aggregates both increased. This indicated that the aggregation interaction between monomers also occurred, in addition to those between monomers and dimers. This may be explained by the difference of side chains on the molecular structure. Besides, the aggregation of R-218 presented a stronger concentration dependence than R-24:1, which may be caused by the relatively poorer solubility of R-218.

### 2.2. The Rheology of High Concentration Solutions

#### 2.2.1. Viscosity of the Three Dye Solutions

**Figure 4:**
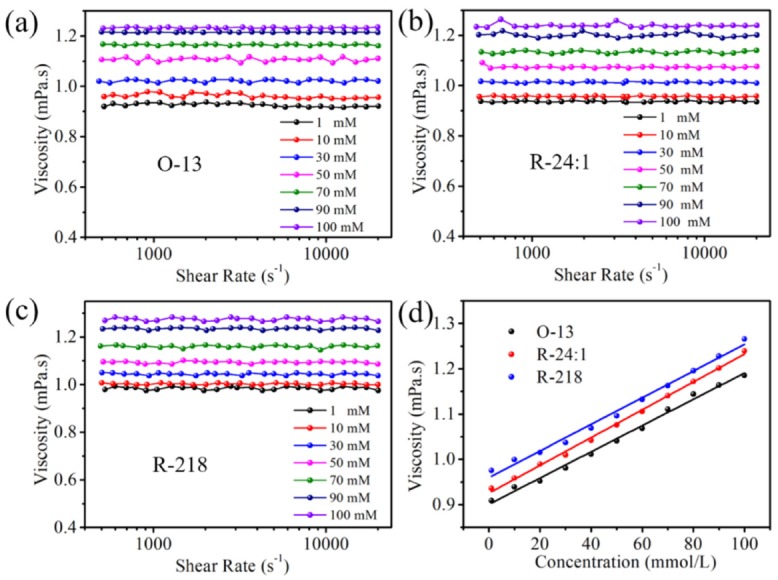
(**a**–**c**) The viscosity of the three dye solutions with different concentrations and shear rate, (**d**) the concentration dependence of viscosity.

The viscosity of the solutions with different concentrations was investigated at different shear rates. As shown in Figure 4, for each dye with a certain concentration, the viscosity remained stable during variation of the shear rates, indicating that all the solutions belong to Newtonian fluid. However, the viscosity displayed concentration dependence; i.e., it increased linearly with the concentration, as shown in Figure 4d. Therefore, it could be inferred that dye aggregation also has an effect on the viscosity. Among the three dyes solutions at the same concentration, the R-218′s viscosity is the largest, which may be caused by the large molecular weight (852.15) and strong aggregation. O-13 is the smallest, which may be due to the small molecular weight (762.02). Therefore, the viscosity could be modified by control the molecular aggregation.

#### 2.2.2. Surface Tension of the Three Dye Solutions

**Figure 5:**
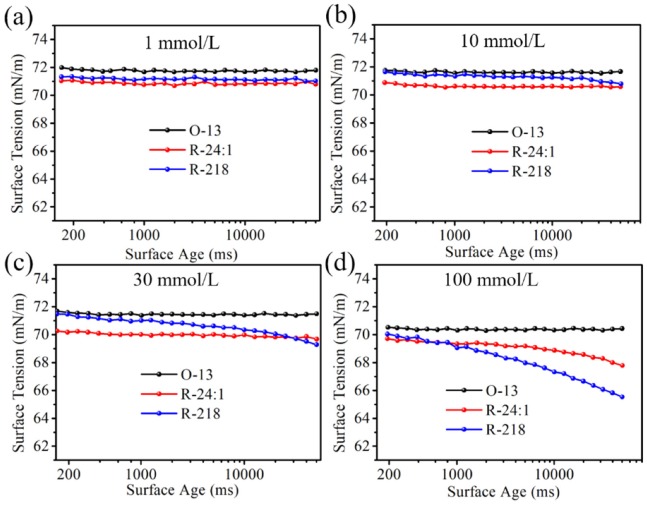
Surface tensions of the dye solutions with different concentrations under different surface ages, (**a**) 1 mmol/L, (**b**) 10 mmol/L, (**c**) 30 mmol/L and (**d**) 100 mmol/L.

The surface tension of the three dye solutions were studied using the Maximum Bubble Pressure Method. As shown in Figure 5, the surface tensions of O-13 solutions hardly changed within the bubble life under the certain solution concentration, and it slightly decreased with the increasing concentration, which indicated that aggregations of the O-13 molecule had little effect on the solutions’ surface tension. This may be caused by the position of hydrophilic sulfonic acid group. As shown in Figure 2, the sulfonic acid groups of O-13 located on the either side of the molecule in the stable configuration, which made it difficult for the molecules to join together to form two phase structure—one side is hydrophobic and the other side is hydrophilic—even if the molecules aggregated together at high concentrations. Therefore, O-13 had little ability to reduce the surface tension of the solution. The surface tension of R-24:1 remained almost constant when the concentration was less than 30 mM, and slightly increased when the concentration was over 30 mM. However, the surface tension of R-218 presented obvious bubble life dependence when the concentration was over 30 mM. These variation trends might be caused by molecular structure and aggregation state. As shown in Figure 2, hydrophilic groups of R-24:1 and R-218 are located on one side of the molecular in the stable configuration. This kind of structure is favorable for the formation of two-phase structure with the increase of aggregations, which would affect the surface tension of the solution. Therefore, R-218 showed more obvious concentration dependence than R-24:1, due to the significant aggregation change of R-218. Therefore, it is crucial to take into consideration the effect of the dye itself on the surface tension when using dyes’ solutions.

## 3. Materials and Methods

All reactive dyes used in this experiment are monochlorotriazine dyes. Three reactive dyes including C.I. Reactive Orange 13 (O-13), C.I. Reactive R218 (R-218), and C.I. Reactive Red 24:1 (R-24:1) were procured from Taiwan Yongguang (Taiwan, China). All dyes are desalted. The molecular structures of dyes are shown in Figure 1. In addition, the solvent used to dissolve the dye in the experiment was ultra-pure water which prepared by Milli-Q^®^ Direct-Q^®^ 8 UV ultra-pure water system (Millipore, Boston, MA, America).

All dyes solutions were prepared in 50 mL volumetric flasks. First, we configured an aqueous dye solution with concentration of 100 mM. A certain amount of dye was accurately weighed through a precision electronic balance. The weighed dye was placed in a 50 mL beaker with a small amount of ultra-pure water and slowly dissolved by a MR Hei-Tec digital magnetic stirrer (Heidolph, Schwabach, Nuremberg, Germany) at 25 °C. Then, we poured the completely dissolved dye into a 50 mL volumetric flask and added ultra-pure water to 50 mL. Finally, we diluted concentrations to 50 mM, 20 mM, 10 mM, 1 mM, 0.1 mM, 0.01 mM.

UV-Vis absorption spectra were recorded by a UV-Vis spectrophotometer U-3900H (Tokyo, Japan) developed by Hitachi Co., Ltd., at 25 °C. Gas-tight quartz cuvettes with 0.01 mm, 0.1 mm and 10 mm path length were used. We checked the influence of the dye concentration on the peak shape; the position of absorption bands corresponding to monomer and aggregate did not exceed the instrumental error.

The dynamic surface tension of reactive dyes solutions was studied by the maximum bubble pressure method. The data were recorded by a Bubble Pressure Tensiometer BP100 (Hamburg, Germany) developed by KRüSS Co. The surface tension of the solutions was measured under different bubble lifetimes. The bubble lifetime refers to the time that from the formation of a bubble at the capillary head to the radius of the bubble equal to the radius of the capillary, which ranges from 1 × 10^2^ ms to 5 × 10^4^ ms. All samples were measured at 25 °C.

Viscosities of the solutions were measured at different shear rates ranged from 5 × 10^2^ s^−1^ to 2 × 10^4^ s^−1^ by a microfluidic visual rheometer FLUDICAM RHEO (Toulouse, France) developed by Formulaction company. All measurements were conducted at 25 °C.

## 4. Conclusions

In this work, the molecular aggregations of the dyes in concentrated solutions were investigated by comparing three typical kinds of reactive dyes. The small group size and linking position of non-conjugate side groups of O-13 resulted in the strongest molecular aggregation in the concentrated solutions, while the better solubility of R-24:1 resulted in weaker aggregation than R-218 in the solutions. The aggregation and the position of hydrophilic group of R-218 reduced the surface tension of the solution and had no effect on viscosity. Therefore, these results will provide support for the research of properties of highly concentrated dyes’ solution.

## Figures and Tables

**Table 1:** The rations of the component curves relative to the absorption spectra.

Dyes	Concentrations (mM)	Curve 1 (%)	Curve 2 (%)	Curve 3 (%)	Curve 4 (%)
O-13	1	33.2	31.8	14.4	12.6
	10	32.0	30.0	16.0	14.4
	100	30.6	28.8	17.5	16.0
R-24:1	1	40.8	38.1	17.4	
	10	40.4	38.1	17.5	
	100	39.4	38.5	18.1	
R-218	1	41.4	37.8	16.6	
	10	39.2	39.9	17.0	
	100	34.0	42.8	18.6	
